# Peak Power Output Is Equivalent to Peak Pulmonary Oxygen Consumption

**DOI:** 10.1111/apha.70238

**Published:** 2026-05-03

**Authors:** Xinyu Liu, Meihan Guo, Zihao Huang, David Montero

**Affiliations:** ^1^ Faculty of Medicine, School of Public Health Hong Kong University Hong Kong SAR Hong Kong; ^2^ Department of Medicine Beth Israel Deaconess Medical Center, Harvard Medical School Boston Massachusetts USA; ^3^ Department of Medicine, School of Clinical Medicine Hong Kong University Hong Kong SAR Hong Kong; ^4^ Libin Cardiovascular Institute of Alberta University of Calgary Calgary Alberta Canada

**Keywords:** aerobic capacity, cardiopulmonary testing, exercise efficiency, large population studies, maximal power output, sex differences

Peak O_2_ consumption ($\dot{V}O_{2}$ peak) elicited by incremental exercise is considered the gold standard of cardiorespiratory fitness and a reliable predictor of mortality in humans [1]. The measurement of $\dot{V}O_{2}$ peak requires a metabolic cart including sensible pieces of equipment, including flow sensors, O_2_/CO_2_ analyzers and moisture removal/drying systems, and large discrepancies in O_2_ consumption are observed within and between metabolic carts from distinct manufacturers. In contrast, peak power output (Wpeak) is easier to measure [2]. A strong linear correlation between Wpeak and $\dot{V}O_{2}$ peak was observed in trained cyclists (*r* ≥ 0.96, *p* < 0.001) [3]. Provided that a similarly close correlation exists in the general population, the question arises whether the measurement of $\dot{V}O_{2}$ peak is indispensable to determine cardiorespiratory fitness. To date, no study has addressed this question in the largest fraction of the adult population, i.e., sedentary or moderately active men and women. We surmised that most researchers assumed that in the adult population at large, there cannot be a strong correlation between Wpeak and $\dot{V}O_{2}$ peak due to moderating factors such as sex, age, fitness status, as well as interindividual differences in the contribution of anaerobic energy production.

Healthy men (*n* = 214) and women (*n* = 207) matched by age (age = 42.3 ± 18.4 vs. 41.3 ± 18.2 year, *p* = 0.565) and endurance physical activity (4.8 ± 3.7 vs. 4.5 ± 3.4 h·week^−1^, *p* = 0.385) were recruited. Inclusion criteria comprised healthy status according to health‐clinical questionnaires, resting ECG/echocardiography screening, absence of current medical symptoms and medication, and no history of chronic disease. The study was approved by the Institutional Review Board of the University of Hong Kong (UW 21–401/22–025) and University of Calgary (REB18‐1654). $\dot{V}O_{2}$ peak was determined with a mixing chamber system (KORR Medical, USA) during an established incremental cycle ergometry (Wahoo KICKR, USA) exercise test in our laboratory [4]. Following a warm‐up period at 10–30 W, the workload was progressively increased by 10–30 W increments every 50 s until exhaustion was reached in a total duration of 7–10 min. Data was averaged over 15 s following recent recommendations [5]. The highest average value defined $\dot{V}O_{2}$ peak provided that at least two of the following established criteria were fulfilled: (i) plateau in O_2_ uptake despite increased workload, (ii) age‐predicted HR_peak_ +/− 10 bpm and/or (iii) respiratory exchange ratio > 1. Peak power output (Wpeak) was defined as the highest workload maintained for at least 10 s during the incremental exercise test.

Linear regression analyses (SPSS 26.0, IBM) determined the relationship between Wpeak and $\dot{V}O_{2}$ peak in (i) all individuals, and (ii) subgroups according to binary variables (sex, age, $\dot{V}O_{2}$ peak). The true correlation was defined as the Pearson correlation coefficient (*r*) corrected for the measurement errors (reliabilities) of Wpeak and $\dot{V}O_{2}$ peak: true *r* = observed *r* ÷ (sqrt (reliability of Wpeak × reliability of $\dot{V}O_{2}$ peak)). The reliabilities of Wpeak and $\dot{V}O_{2}$ peak were determined by the *r* of duplicate (test–retest) measurements performed in 10 study participants (50% ♀) within 2–7 days. The impact of sex, age and cardiorespiratory fitness ($\dot{V}O_{2}$ peak/kg) on the relationship between Wpeak and $\dot{V}O_{2}$ peak was assessed via the comparison of the regression line slopes in these subgroups: men vs. women, young vs. older, high vs. low $\dot{V}O_{2}$ peak/kg. Finally, the dependent sample *t*‐test compared exercise economy ($\dot{V}O_{2}$/W) at moderate and peak exercise intensities. The percentage difference between exercise economy (O_2_ consumption ($\dot{V}O_{2}$)/Watt (W)) at moderate (100 W) and peak exercise intensities was determined to assess the anaerobic energy contribution to Wpeak.

Wpeak and $\dot{V}O_{2}$ peak were linearly related (*r* = 0.93, *p* < 0.001) (Figure 1). The measurement errors (reliabilities) in Wpeak and VO_peak_, as determined by the correlation of duplicate (test–retest) measurements, were *r* = 0.99 and *r* = 0.97, respectively. The true correlation between Wpeak and $\dot{V}O_{2}$ peak, i.e., correcting for measurements errors in Wpeak and $\dot{V}O_{2}$ peak, approached a perfect linear relationship (*r* = 0.95, *p* < 0.001). The linear regression slopes did not differ according to sex (*p* = 0.064), age (*p* = 0.787) and $\dot{V}O_{2}$ peak/kg (*p* = 0.539). The $\dot{V}O_{2}$/W ratio was higher at moderate (100 W) compared with peak exercise intensities (12.1 ± 2.5 vs. 11.6 ± 1.6 mL·min^−1^·W^−1^, *p* < 0.001). The percentage difference in $\dot{V}O_{2}$/W between moderate and peak exercise intensities, reflecting the anaerobic energy percentage contribution to Wpeak, was 4.5%.

**FIGURE 1:**
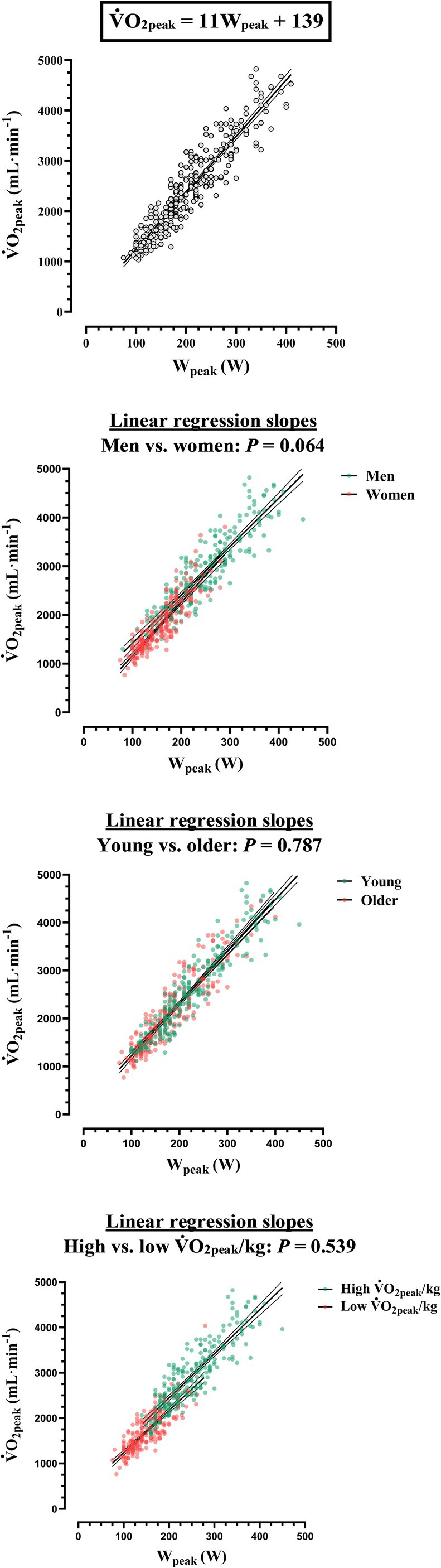
Relationship between peak power output (Wpeak) and peak O_2_ consumption ($\dot{V}O_{2}$ peak) and impact of sex, age and cardiorespiratory fitness. The line of best fit (regression line) with the 95% confidence interval is displayed in the background. The linear regression slopes are compared as follows: Men vs. women, young vs. older, high vs. low $\dot{V}O_{2}$ peak.

If the relationship between two independently measured variables results in a perfect linear correlation (*r* = 1), both can be used interchangeably. In the medical field, the relationship between surrogate variables is typically far from a perfect linear correlation due to three main sources of variability: (1) measurement errors, (2) intrinsic differences in what each measurement precisely quantifies, and (3) biological variability of the measured variables. As such, the linear relationship between Wpeak and $\dot{V}O_{2}$ peak in the general population is striking (Figure 1). When the potential impact of measurement errors is removed, the linear correlation is even better (*r* = 0.95), and allows for $\dot{V}O_{2}$ peak of the general population to be described as:

$$\dot{V}O_{2}\;\text{peak}\;\left(\mathrm{mL}\cdot \min^{-1}\right)=11\text{Wpeak}+139$$

The slope of the linear equation yields a cycling exercise efficiency (CEE) of 26.2%, based on the equation of Davis et al. (CEE = (∆W/∆ $\dot{V}O_{2}$) × 288) [6]. This CEE value falls within the expected range (25.7%–28.2%) for cycle ergometry incremental exercise protocols of medium‐to‐long duration (9–16 min) [7]. CEE can be specifically adjusted to the duration of the cycle ergometry protocol to maximize accuracy [7].

Age and cardiorespiratory fitness do not influence the relationship between Wpeak and $\dot{V}O_{2}$ peak, which contradicts typical assumptions in Exercise Physiology textbooks. How can aging and fitness status not modulate such a relationship? Their influence is indeed observed in studies measuring exercise efficiency on a treadmill [8]. Instead, with cycle ergometry, neither sex, aging or fitness affect CEE [9]. This divergence can be explained by the confounding biomechanical factors affecting running, but not cycling, exercise efficiency [10], and indicates that the ‘neat’ (metabolic) relationship between Wpeak and $\dot{V}O_{2}$ peak is robust to major moderating factors in humans.

In conclusion, Wpeak and $\dot{V}O_{2}$ peak are statistically equivalent in healthy women and men and linearly correlated. Major potential moderating factors such as sex, age and cardiorespiratory fitness do not modify the relationship between Wpeak and $\dot{V}O_{2}$ peak. Furthermore, the relationship of O_2_ consumption and power output during incremental exercise denotes no relevant confounding influence of anaerobic energy production at peak effort. Collectively considered, large population studies can rely on incremental exercise Wpeak as a robust and accurate measure of cardiorespiratory fitness.

## Author Contributions

**Meihan Guo:** investigation, writing – review and editing, formal analysis, supervision. **David Montero:** conceptualization, investigation, funding acquisition, writing – original draft, writing – review and editing, validation, methodology, formal analysis, supervision, data curation, resources. **Xinyu Liu:** investigation, formal analysis, supervision, writing – review and editing. **Zihao Huang:** investigation, writing – review and editing, formal analysis.

## Funding

Research Grant Council of Hong Kong (106210224 and 104006024, to D.M.).

## Conflicts of Interest

The authors declare no conflicts of interest.

## Data Availability

The data that support the findings of this study are available from the corresponding author upon reasonable request.
